# Effect of artesunate and relation with TGF-β1 and SMAD3 signaling on experimental hypertrophic scar model in rabbit ear

**DOI:** 10.1007/s00403-019-01960-7

**Published:** 2019-08-09

**Authors:** Xiaolin Nong, Girju Rajbanshi, Ling Chen, Jiaquan Li, Zhan Li, Taotao Liu, Shihai Chen, Gao Wei, Jushang Li

**Affiliations:** 1 Department of Oral and Maxillofacial Surgery, College and Hospital of Stomatology, Guangxi Medical University, 5-715, No. 10 Shuangyong Road, Nanning, 530021 Guangxi China; 2 Department of Pediatrics Dentistry and Preventive Dentistry, College and Hospital of Stomatology, Guangxi Medical University, No. 10 Shuangyong Road, Nanning, 530021 Guangxi China; 3 Medical Science Research Center, Guangxi Medical University, No. 22 Shuangyong Road, Nanning, 530021 Guangxi China; 4 Department of Pharmacy, The First Affiliated Hospital of Guangxi Medical University, No. 6 Shuangyong Road, Nanning, 530021 Guangxi China; 5 Department of Cosmetics and Plastic Surgery, The First Affiliated Hospital of Guangxi Medical University, No. 6 Shuangyong Road, Nanning, 530021 Guangxi China; 6 Department of Dermatology, The First Affiliated Hospital, Guangxi Medical University, No. 6 Shuangyong Road, Nanning, 530021 Guangxi China

**Keywords:** Artesunate, Hypertrophic scar, SMAD3, TGF-β1

## Abstract

Artesunate (ART) is the derivative of artemisinin isolated from the traditional Chinese medicine *qinghao*. Although several studies reported the efficiency of artesunate in the treatment of malaria, inhibiting fibroblasts and collagen synthesis, the association between artesunate and scar formation is unclear. The research was designed to study the significance of artesunate (ART) on the expression of transforming growth factor (TGF-β1) and small mother against decapentaplegic (SMAD3) in rabbit's ear hypertrophic scar model. Twenty-four New Zealand white rabbits were randomly divided into six groups: control group, matrix group, low-concentration artesunate group (0.48%), medium-concentration artesunate group (0.96%), high-concentration artesunate group (1.92%) and silicone gel group. Punched defects were established on each rabbit’s ear which resulted in a hypertrophic scar. On the 28th day, topical artesunate creams were applied twice a day except on the control group. On the 56th day, scar samples were collected for histopathology and immunoassay. Hematoxylin and eosin staining, Van Gieson staining, immunohistochemistry and Western blot analysis were done. Amongst the six groups, findings showed that the medium-concentration artesunate group (0.92%) efficiently decreased hypertrophic scar formation and significantly reduced fibroblasts and collagen synthesis. The results had also shown a decrease in the expression of transforming growth factor (TGF-β1) and declined small signal mother against decapentaplegic (Smad3). The overall study shows efficacy and mechanism of artesunate. It concluded that the medium concentration of artesunate (0.92%) could be an effective therapeutic agent for hypertrophic scars.

## Introduction

A hypertrophic scar is a clinical manifestation of malfunctioned wound healing process identified by an overabundance of collagen and extreme deposition of extracellular matrix which is caused by physical injury, inflammatory reactions and deep burn [8]. Hypertrophic scar results in the loss of tissue function and disfigurement [24, 29]. Currently, treatment of hypertrophic scar during an abnormal wound healing process has been a great challenge for medical practitioners [9]. Previously, many alternative therapeutic modalities such as avotermin, intralesional steroid injection, onion extract, and heparin gel application, silicone gel sheeting, bleomycin injection, and pulsed dye laser had been tested to treat hypertrophic scar occurrence. However, none could decrease or block this process, and no authentic treatment has been identified yet. Therefore, a reliable treatment procedure which provides painless, effective wound healing capacity and patient and physician friendly environment to alleviate hypertrophic scar needs to be discovered [13, 26].

Artesunate (ART) is the derivative of artemisinin isolated from the traditional Chinese medicine *qinghao* and has a favorable pharmacological profile with water solubility and high oral bioavailability [1, 28]. It has been used clinically as anti-malarial agents for many years [20]. Furthermore, ART has been shown to possess antiviral, anti-inflammatory and anti-cancer effects [9, 10, 16, 19]. The ART facilitates hypertrophic scar fibroblast cells to apoptosis in vitro by the change of cell cycle. It also suggested that intracellular calcium variation may be one of the mechanisms of hypertrophic scar fibroblast apoptosis induced by ART [14]. Another study also demonstrated that artesunate could induce the apoptosis of fibroblasts, inhibit TGF-β1-induced epithelial-to-mesenchymal transition and downregulate the expression of TGF-β1 in an animal model [36]. Additionally, the mechanism of ART inhibited the proliferation of fibroblast in keloid may be related to the up-regulation of calcium/calmodulin-dependent serine protein kinase (CASK) and down-regulation of inhibitors of differentiation 1(ID1) [31].

Transforming growth factor (TGF-β1) is a significant signaling factor in wound healing process by regulating cell differentiation, collagen production and extracellular matrix degradation. The prolonged high expression of TGF-β1 is accepted for the formation of hypertrophic scars. Inhibition of TGF-β1 signaling pathway may express one of the effective strategies for limiting excessive scarring [2]. Additionally, TGF-β initiates and terminates tissue repair, and its sustained production contributes to the development of fibrosis. It related to a wide variety of fibrotic diseases affecting many body organs including the liver, kidney, lung, and skin [18]. Recent research suggests that the TGF-β/Smad signaling pathway closely related to a normal scar and hypertrophic scar formation. Moreover, a member of the Smad family mainly SMAD3 interposes collagen production in dermal fibroblasts stimulated by TGF-β. Increasing evidence has presented that blocking TGF-β/Smad signaling pathway prohibits the development of hypertrophic scars [33]. This study was designed to analyze the efficiency of ART cream on hypertrophic scars in vivo, as well as elucidate the mechanism of action of ART, and explore the function of the transforming growth factor (TGF-β1)/small mother against decapentaplegic (Smad3) signaling pathway in hypertrophic scar formation.

## Materials and methods

This experimental study was performed by following the guidelines for the use of laboratory animal subjects in research set by Animal Center of Guangxi Medical University and Hospital of Stomatology affiliated to Guangxi Medical University, license number: SCXK GUI (2009-0002).

### Preparation of drugs and chemicals

#### Preparation of paste for a matrix group

The oil-soluble materials 2.4 g white petroleum, 1.6 g octadecanol, 0.4 g glycerol monostearate were heated in an electric constant-temperature water bath to evaporate the dish to 80 °C. The water-soluble materials 0.2 g sodium lauryl sulfate, 1.4 g glycerin, 0.04 g ethyl *p*-hydroxybenzoate and distilled water were placed in another evaporating dish heated to 80 °C. The aqueous phase was slowly added to the oil phase at a temperature of 80 °C. 20 g of matrix control pastes were prepared.

#### Preparation of ART cream with different concentrations

Artesunate purchased from Guilin Pharmaceutical Co. Ltd. (No. 43 Qilidian Road, Guilin, Guangxi, China 541004). According to the above method, 20 g of matrix control paste was added, and the corresponding weight of artesunate was made. 0.48% low artesunate drug concentration, 0.96% medium artesunate drug concentration, and 1.92% high artesunate drug concentration were prepared.

### Animal research and therapy

Twenty-four New Zealand white rabbits, obtained from Guangxi Medical University animal experiment center in Nanning, China, with an initial body weight of 1.8–2.5 kg, were used. All animals were kept under constant conditions (temperature 25 ± 1 °C) and had free access to a standard diet and drinking water. All animal treatments were strictly following the International Ethical Guidelines and the National Institutes of Health Guide concerning the Care and Use of Laboratory Animals, and the experiments were carried out with the approval of the Animal Experimentation Ethics Committee of Guangxi Medical University animal experiment center.

**Fig. 1:**
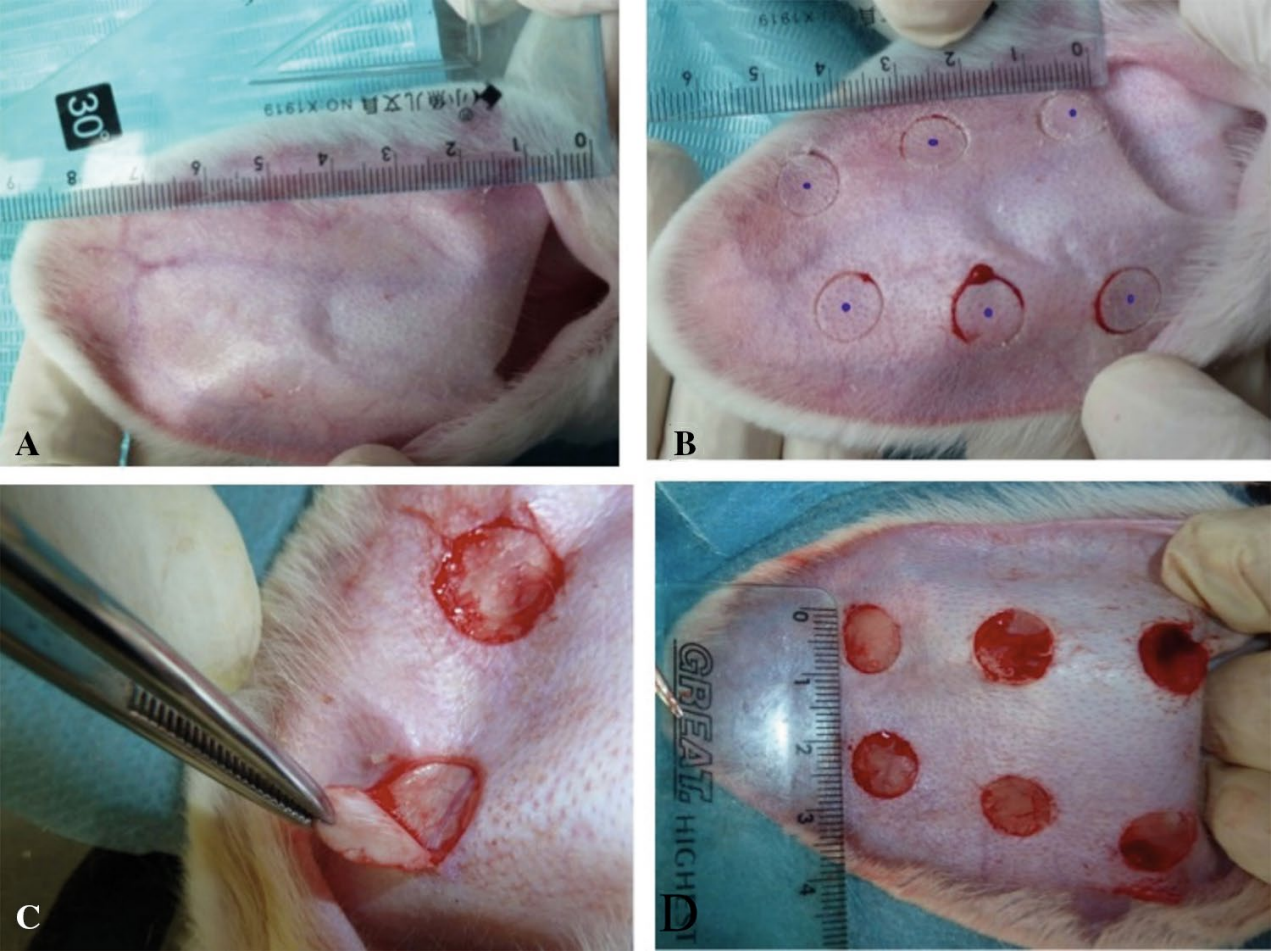
Preparation of hypertrophic scars on the ventral side of the rabbit's ear. Six wounds 1 cm in diameter were created on bare cartilage on the ventral surface of rabbit's ear using dermal punch biopsy defect

Rabbits were randomly distributed into six groups as follows: the control group, matrix group, and three ART groups in a dose-dependent manner (0.48% low-concentration artesunate, 0.96% medium-concentration artesunate, 1.92% high-concentration artesunate), and silicone gel group, four rabbits in each group. Hypertrophic scars were induced on the ventral surface of rabbit ears. Briefly, the rabbits were anesthetized with the administration of 10% chloral hydrates (3–4 ml/kg) and then cycloid wounds 1 cm in diameter were created down to bare cartilage and dislodged the perichondrium on the rabbit's ear, six injuries in each ear (Fig. 1). Each wound was covered by iodophors. Then, after maintaining homeostasis, the wound was enclosed with an antiseptic bandage. The sterile dressing was removed at the end of the 3rd week. The scars of the matrix group were thinly applied with matrix paste cream twice a day; the three ART groups were applied the corresponding ointment with ART twice a day and silicone gel on silicone gel group from 4th week to the end of the 7th week. In the control group, no ointment was applied to rabbits. At the end of 7th week, all animals were anesthetized and killed, and then each of the hypertrophic scars tissues was divided for further laboratory investigation as follows: 10% formaldehyde for histopathological examination, Van Gieson (VG), immunohistochemical analyses for TGF-β1 and Smad3 protein expression, and Western blot analysis.

### H&E and determination of Scar Elevation Index

Scar tissues were embedded in paraffin and cut into 4-μm sections for H&E and Van Gieson (VG) staining. H&E was staining performed. Briefly, the tissue sections were stained in hematoxylin dye, washed in running water, stained using eosin and finally mounted under coverslips. Then slides were studied using an ordinary optical microscope from Japan Olympus Corporation. The pathological features were observed and the degree of scar hyperplasia was expressed as SEI. SEI is defined as the ratio of the scar tissue height to the healthy tissue below the hypertrophic scar. Hypertrophy Index (HI), numerical densities of fibroblasts on the area (NA) and area density of collagen fibers (AA) were also analyzed.

### Van Gieson staining

Van Gieson staining is the simplest method of differential staining of collagen and other connective tissue. Briefly, the tissue was dewaxed, rehydrated, stained in Verhoeff’s solution for 1 h, washed and then differentiated in 2% ferric chloride for 2 min. Tissue should be completely black microscopically and then treated with 5% sodium thiosulfate for 1 min, washed and counterstained in Van Gieson's solution for 3–5 min. Finally, the tissue was dehydrated, cleared and sealed. The Image Pro plus 4.5 software evaluated collagen deposition.

### Immunohistochemical analysis

Immunostaining techniques were performed using the scar tissue to demonstrate the presence of TGF-β1 and SMAD3. The sections were placed on slides and deparaffinized for 5 min. Each in the three-step xylene series rehydrated using a set of graded alcohol and distilled water. Heating was applied for 20 min o induce antigen retrieval in 10 mM sodium citrate buffer, pH 6.0. Endogenous peroxide activity was quenched using 3% hydrogen peroxide in absolute methanol for 7 min at room temperature (RT). The tissue sections were rinsed thrice with PBS (pH 7.4) for 5 min between each consecutive step. The parts were then incubated in blocking serum for 5 min to prevent nonspecific antibody binding. After that, the sections were incubated with a primary antibody for 60 min in a humidity chamber at RT. After treating the articles with biotin-labeled secondary antibody for 15 min and streptavidin-peroxidase enzyme for 15 min at RT, the color reaction was performed using aminoethyl carbazole (AEC) chromogen (ThermoFisher Scientific, USA) for 5–10 min. Sections were counterstained with Mayer’s hematoxylin for 1–2 min and suspended in a water-based mounting medium (ThermoFisher Scientific, USA). A double-blind method at high magnification (400 ×) was used to get photographs of samples and stored in the computer, then Image Pro Plus (IPP) Version 6.0 professional image analysis software (Media Cybernetics, USA) was used.

### Western blot analyses

The scar tissue was homogenized in cell lysis buffer for Western blot. The protein lysates were processed from cultured fibroblasts separated by 8 or 10% polyacrylamide gel, transmitted to a polyvinylidene difluoride membrane, and immunoblotted with primary antibodies at 4 °C overnight. The membranes were incubated with horseradish peroxidase-conjugated secondary antibodies. The primary antibodies used were mouse monoclonal anti-TGF-β1 and mouse monoclonal anti-Smad3 obtained from Santa Cruz Biotechnology (Santa Cruz, Calif., USA) and mouse monoclonal glyceraldehyde 3-phosphate dehydrogenase (GAPDH) antibody obtained from Cell Signaling Technology (Beverly, Mass., USA). GAPDH protein was used as an internal control. Visualization of the detection system (Millipore) was followed by exposure to X-ray film. The protein bands were then analyzed by Image J software. The gray-scale values of detected proteins were normalized to the benefits of the corresponding GAPDH band to determine the expression change of the protein.

### Statistical analysis

Statistical results were achieved by the SPSS software version 13.0 for Windows and data were given as means ± standard deviation (SD). Comparison was by one-way ANOVA; *P* < 0.05 was considered to be statistically significant for experimental and control groups.

## Results

### Effect of ART cream on hypertrophic scars in rabbit's ear

**Fig. 2:**
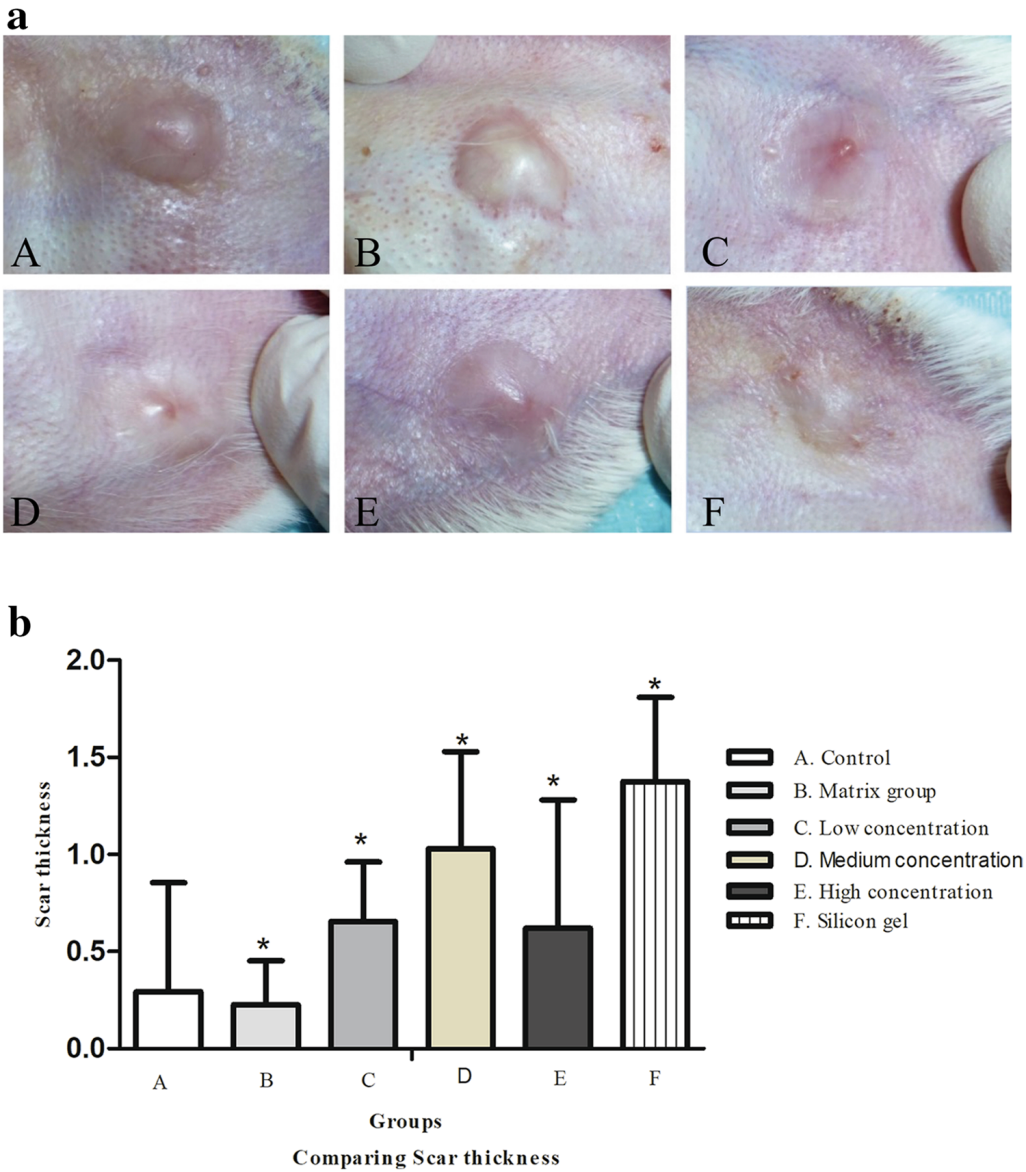
**a** Macroscopic hypertrophic scar view on rabbit's ear observed on day 28 after medication. (A) Control group, (B) matrix group, (C) low-concentration ART cream, (D) medium-concentration ART cream, (E) high-concentration ART cream, and (F) silicone gel group. Scar on medium-concentration ART cream (D) and silicone gel groups (F) was smooth, and the color is close to normal. **b** Comparison of the scar thickness in the rabbit's ear scar samples. (A) control group, (B) matrix group, (C) low-concentration ART cream, (D) medium-concentration ART cream (E) high-concentration ART cream, and (F) silicone gel group. Error bars represent SD. A column represents mean ± SD, *n* = 4 *P < 0.05 compared with control group

Three weeks after the operation, a raised and visible scar gradually initiated in a primary wound healing stage. At the end of the 7th week, appearance of representative wounds in each group was assessed (Fig. 2a), which displayed that hypertrophic scars from the control and matrix group were highly elevated from surrounding tissue, while injuries in the treatment groups were flatter than those in the control and matrix groups. There was a significant difference between the control group and treatment groups (**P < 0.05) (Fig. 2b).

### H&E and VG staining findings

**Fig. 3:**
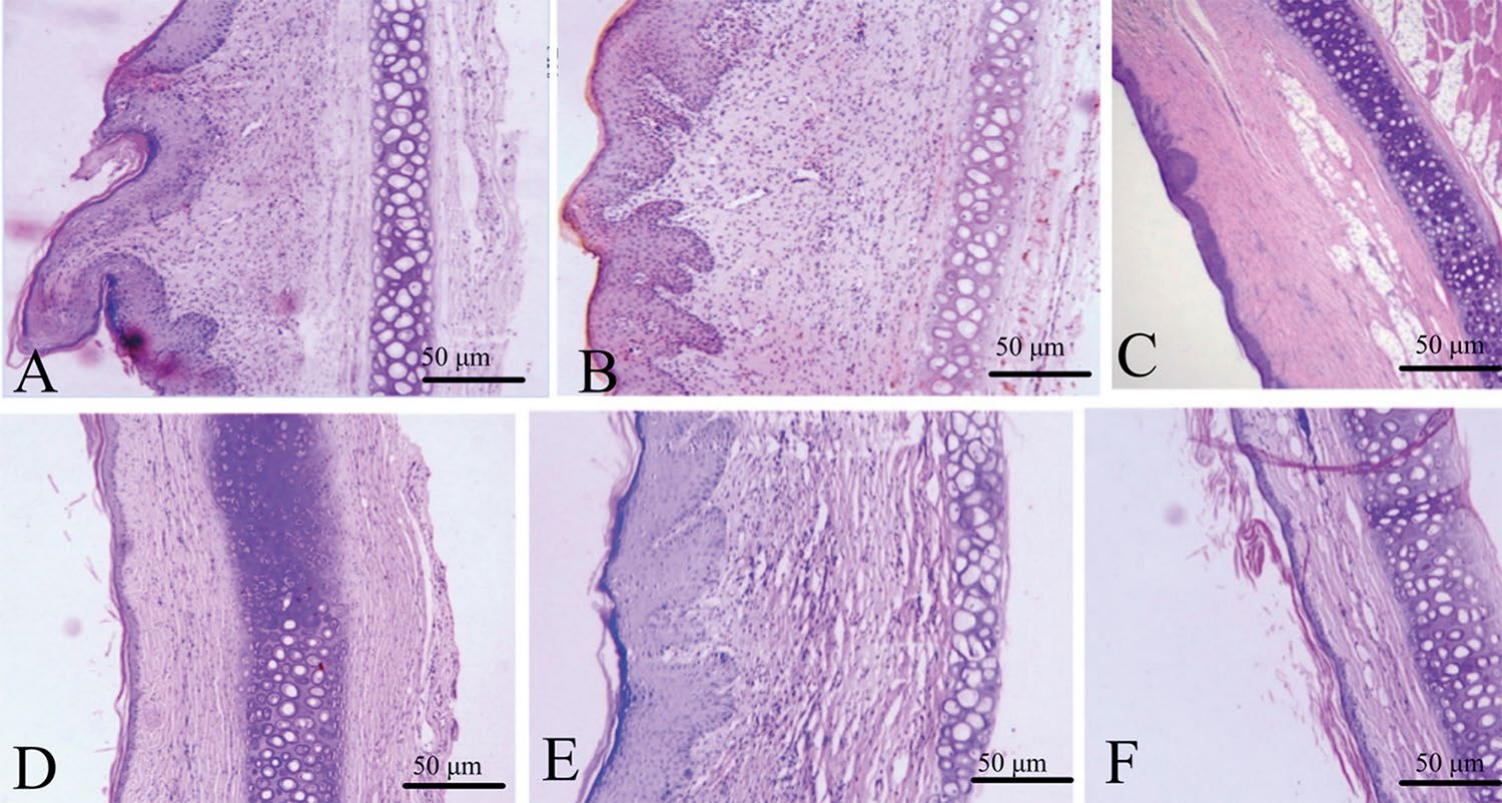
H&E staining for histological analysis of hypertrophic scar on rabbit's ear. **a** Control group, **b** matrix group, **c** low-concentration ART cream, **d** in medium-concentration ART cream, **e** high-concentration artesunate cream, and **f** silicone gel group. The scale bar on H&E 50 µm

**Fig. 4:**
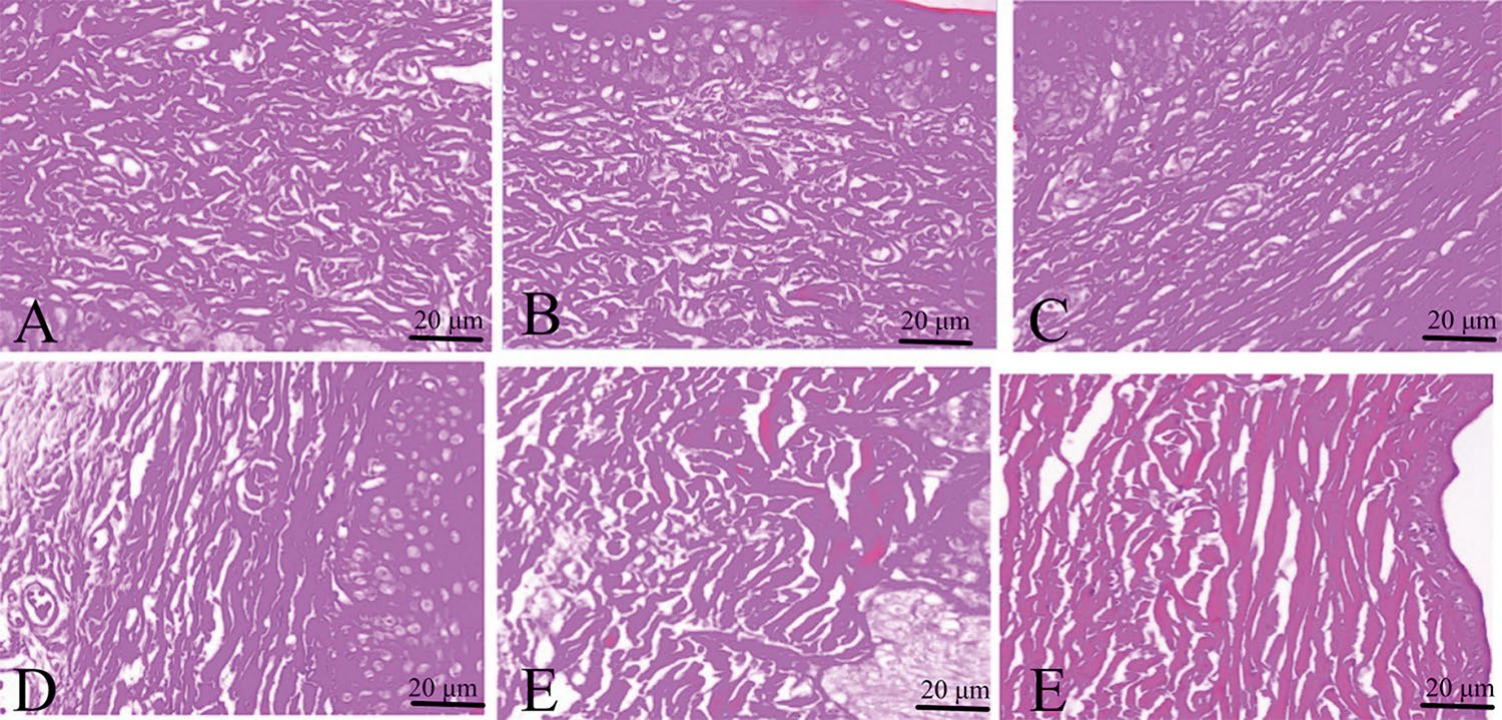
Van Gieson (VG) staining for collagen fibers on hypertrophic scar of rabbit's ear. **a** Control group, **b** matrix group, **c** low-concentration ART cream, **d** medium-concentration ART cream, **e** high-concentration ART cream, and **f** silicone gel group. Scale bar 20 µm

Hematoxylin–eosin staining showed dermis layer with ridges, angiogenesis and irregular fibroblasts in control group and matrix groups; however, this ameliorated in the ART treatment groups and silicone gel group (Fig. 3). Figure 4 shows typical rabbit ear scar sections stained with Van Gieson (VG) staining. The collagen fibers were denser, thicker and more disordered in control and matrix groups. Typical bundle-shaped collagen fibers appeared in the ART treatment group, especially in the medium-concentration artesunate group, while there was a marked improvement in silicone gel groups with less density and arranged collagen fibers.

### Hypertrophic Index (HI), fibroblast number density (NA), and the surface density of collagen fibers (AA)

**Fig. 5:**
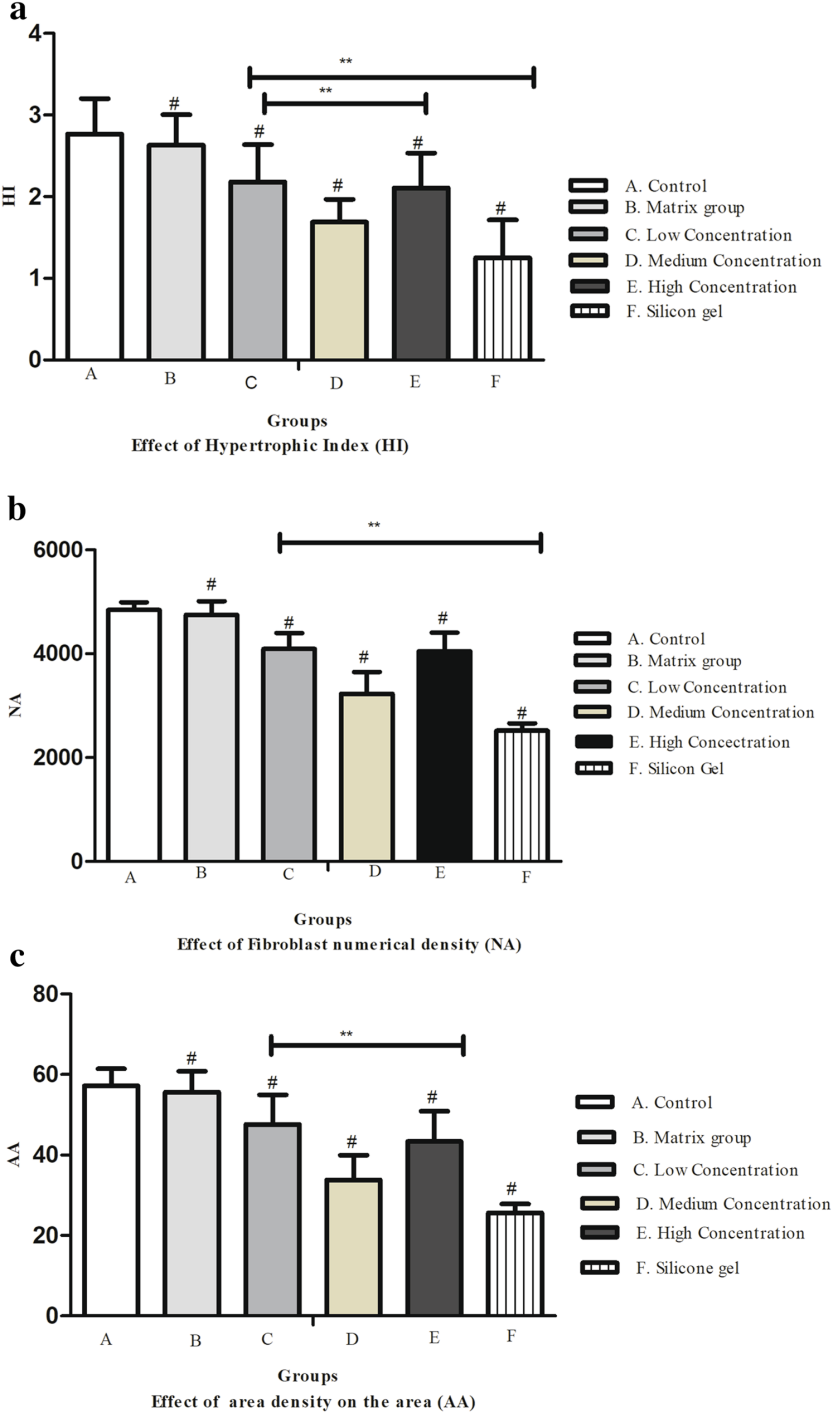
**a** Hypertrophic Index (HI) on rabbit's ear scar samples. In (A) control group, (B) matrix group, (C) low-concentration ART cream, (D) medium-concentration ART cream, (E) high-concentration ART cream, and (F) silicone gel group. Error bars represent SD. A column represents mean ± SD, *n* = 4 **P < 0.01 compared with artesunate treatment group and silicone gel group, ^#^*P* < 0.05 compared with the control group. **b** Fibroblast number density (NA) on the hypertrophic scar on the rabbit's ear. (A) Control group, (B) matrix group, (C) low-concentration ART cream, (D) medium-concentration ART cream, (E) high-concentration ART cream, and (F) Silicone gel group. Error bars represent SD. A column represents mean ± SD, *n* = 4 **P < 0.01 compared with ART treatment group and silicone gel group, ^#^*P* < 0.01 compared with the control group. **c** Surface area of collagen fibers (AA) on the hypertrophic scar on the rabbit's ear. (A) Control group, (B) matrix group, (C) low-concentration ART cream, (D) medium-concentration ART cream, (E) high-concentration ART cream, and (F) silicone gel group. Error bars represent SD. A column represents mean ± SD, *n* = 4 **P < 0.01 compared with A&M treatment group, ^#^*P* < 0.01 compared with control group

Hypertrophic scar index, Fibroblast number density and surface density of collagen fibers were significant between control group, matrix group, and treatment groups, (^#^*P* < 0.05); and artesunate group (low concentration, medium concentration, high concentration) and silicone gel group showed statistical significance (**P < 0.01) (Fig. 5a–c).

### Effect of ART on TGF-β1 protein expression

**Fig. 6:**
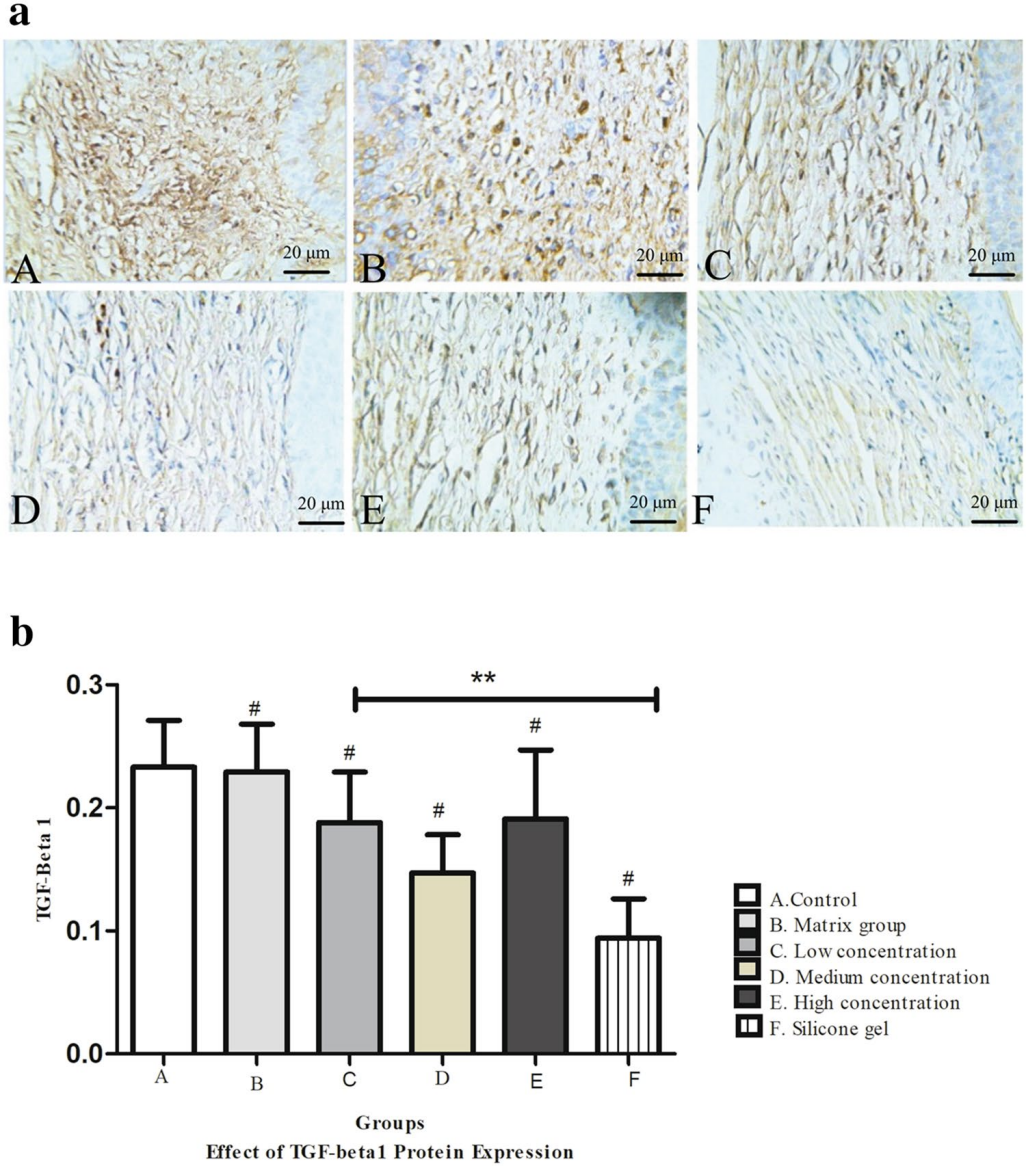
**a** Immunohistochemical findings on TGF-β1 protein expression on rabbit hypertrophic scars for fibroblast on dermis layer. (A) Control group, (B) matrix group, (C) low-concentration ART cream, (D) medium-concentration ART cream, (E) high-concentration ART cream, and (F) silicone gel group. DAB, scale bar, 20 μm. **b** Effect of TGF-β1 protein expression on the hypertrophic scar on rabbit's ear. (A) Control group, (B) matrix group, (C) low-concentration ART cream, (D) medium-concentration ART cream, (E) high-concentration ART cream, and (F) silicone gel group. Error bars represent SD. A column represents mean ± SD, *n* = 4 **P < 0.01 compared with artesunate treatment group, ^#^*P* < 0.01 compared with the control group

Figure 6a shows the expression of TGF-β 1 protein extracted from frozen rabbit ear tissue. The expression of TGF-β 1 protein in the control and matrix group markedly increased, while ART dose-dependent group and silicone gel group decreased. The treatment group and control group were statistically significant (^#^*P* < 0.01) and artesunate treatment group and silicone gel group were also statistical significant (**P < 0.01) (Fig. 6b).

### Effect of ART on SMAD 3 protein expression

**Fig. 7:**
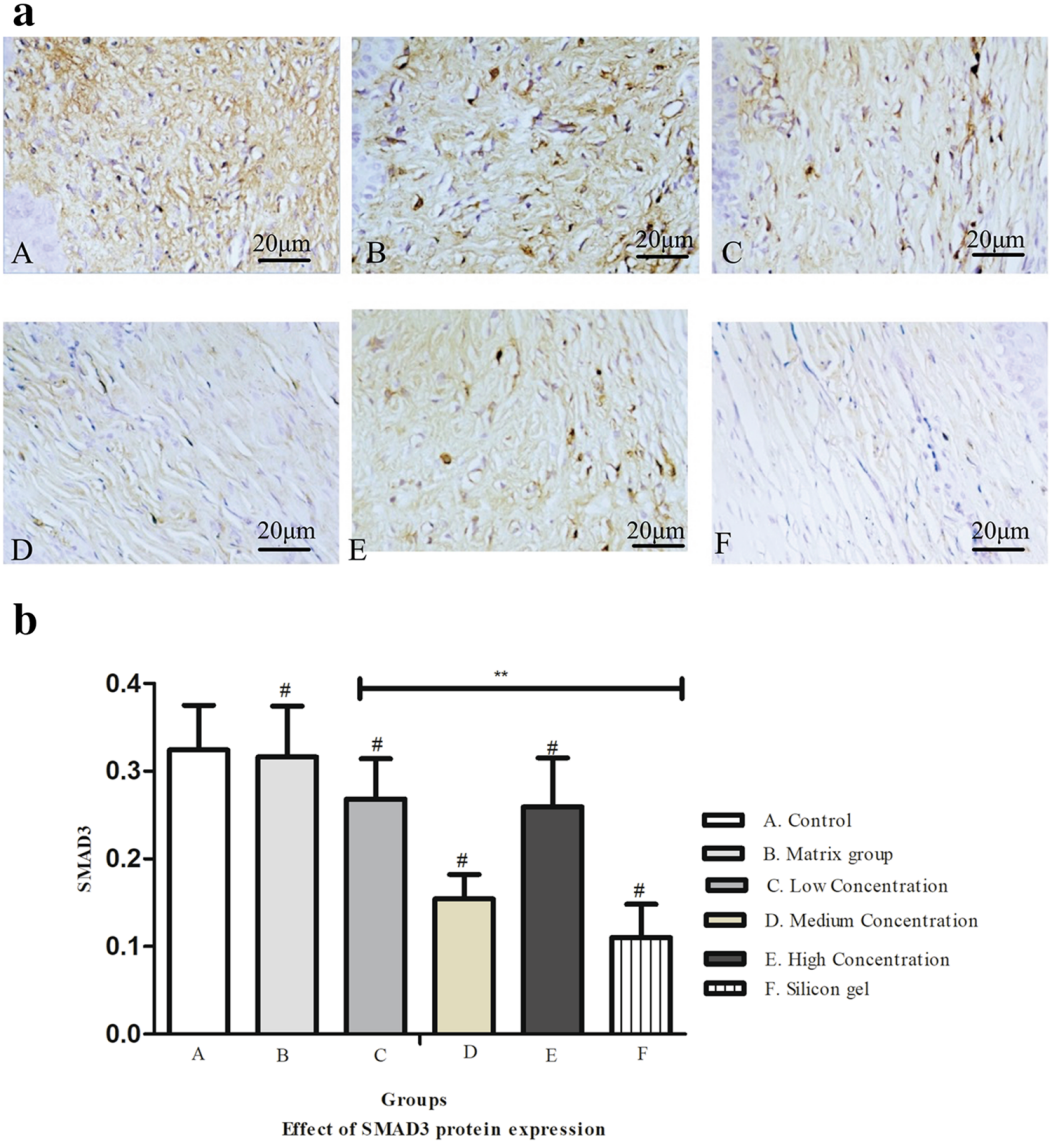
**a** Immunohistochemical findings on SMAD3 protein expression on rabbit hypertrophic scars for fibroblast on dermis layer. (A) Control group, (B) matrix group, (C) low-concentration ART cream, (D) medium-concentration ART cream, (E) high-concentration ART cream, and (F) silicone gel group. DAB, scale bar, 20 μm. **b** Effect of SMAD3 protein expression on the hypertrophic scar on rabbit's ear. (A) Control group, (B) matrix group, (C) low-concentration ART cream, (D) medium-concentration ART cream, (E) high-concentration ART cream, and (F) silicone gel group. Error bars represent SD. A column represents mean ± SD, *n* = 4 **P < 0.01 compared with artesunate treatment group, ^#^*P* < 0.01 compared with the control group

Figure 7a shows the expression of SMAD3 protein extracted from frozen rabbit ear tissue. The expression of SMAD3 protein in the control and matrix groups markedly increased, while decreased in the ART dose-dependent group and silicone gel group. The treatment group and control group were statistically significant (^#^*P* < 0.01) and artesunate treatment group and silicone gel group were also statistically significant (**P < 0.01) (Fig. 7b).

### Western blot detection of TGF-β1 protein expression and SMAD 3 protein expression

**Fig. 8:**
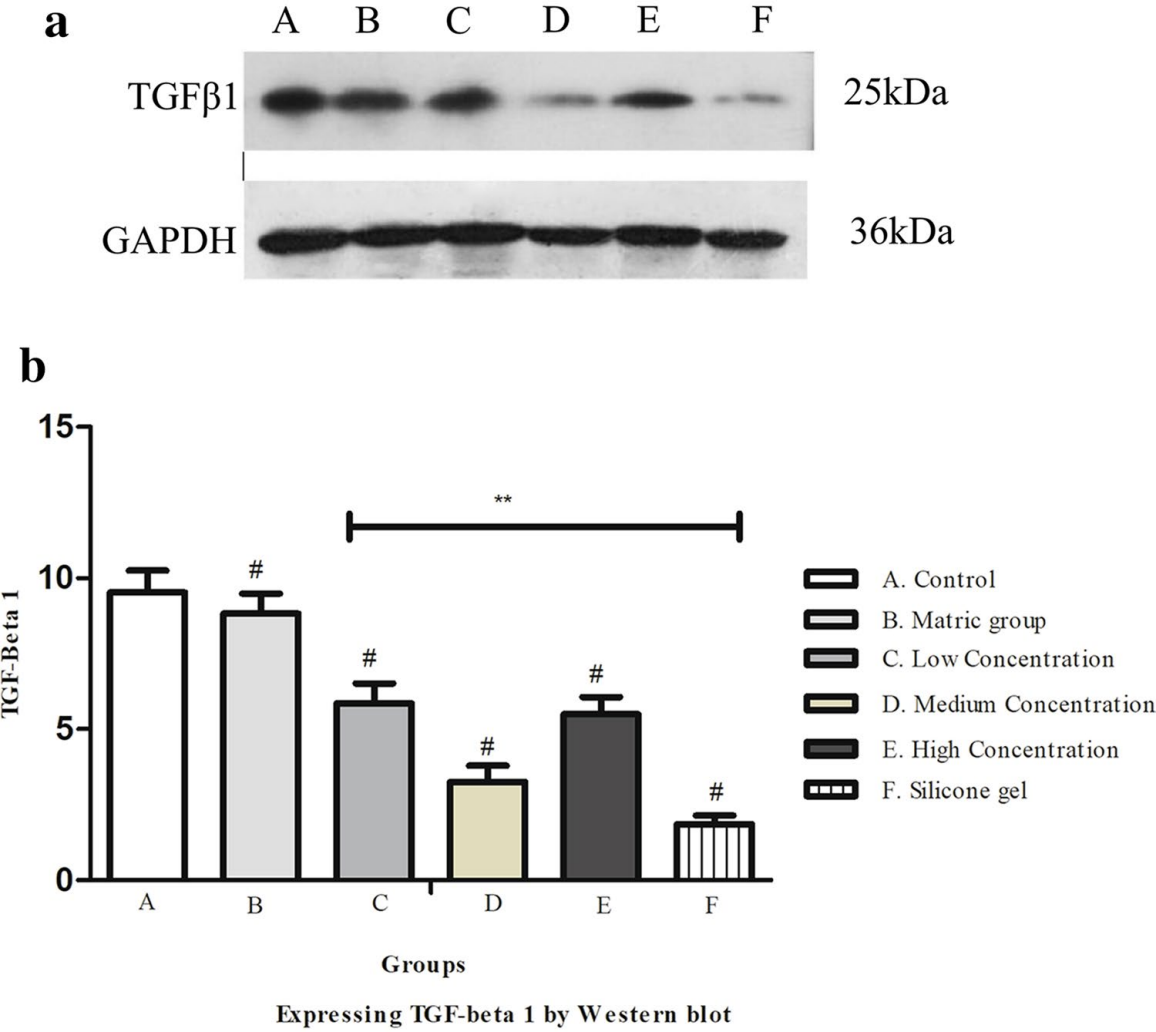
**a** Effects of ART cream on TGF-β1 protein expression analyzed by Western blot using anti-TGF-β1 and anti-GAPDH antibodies. The intensities of TGF-β1 bands normalized to GAPDH of the corresponding treatment groups. (A) Control group, (B) matrix group, (C) low-concentration ART cream, (D) medium-concentration ART cream, (E) high-concentration ART cream, and (F) silicone gel group. **b** Western blot expressing TGF-β1 on the hypertrophic scar on rabbit's ear. (A) Control group, (B) matrix group (C) low-concentration ART cream, (D) medium-concentration ART cream, (E) high-concentration ART cream, and (F) silicone gel group. Error bars represent SD. A column represents mean ± SD, *n* = 4 **P < 0.01 compared with ART treatment group, ^#^*P* < 0.01 compared with control group

**Fig. 9:**
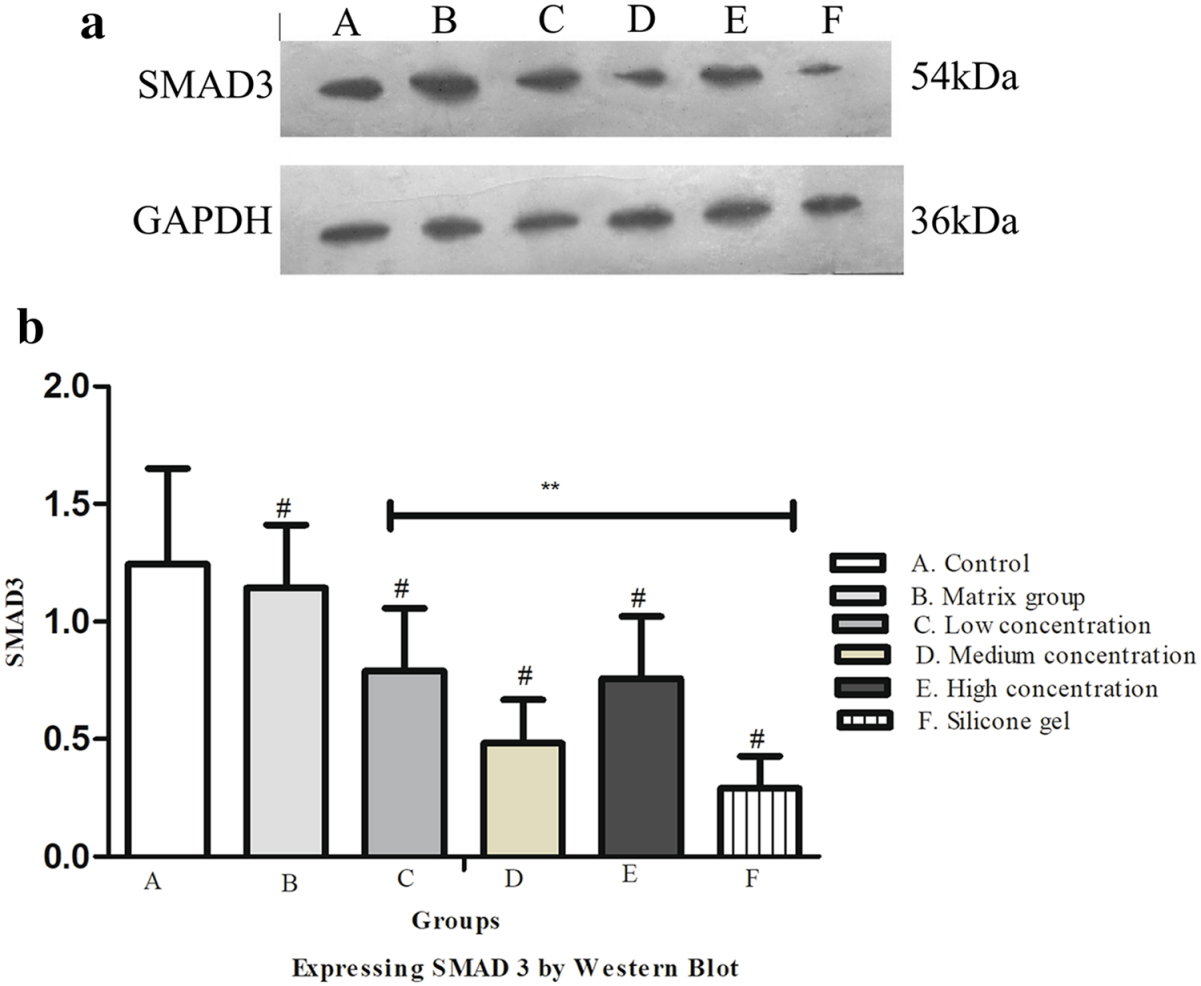
**a** Effects of ART cream on Smad3 protein expression analyzed by Western blot using anti-Smad3 and anti-GAPDH antibodies. The intensities of Smad3 bands normalized to GAPDH of the corresponding treatment groups. (A) Control group, (B) matrix group, (C) low-concentration ART cream, (D) medium-concentration ART cream, (E) high-concentration ART cream, and (F) silicone gel group. **b** Western blot expressing SMAD3 on the hypertrophic scar on rabbit's ear. (A) Control group, (B) matrix group, (C) low-concentration ART cream, (D) medium-concentration ART cream, (E) high-concentration ART cream, and (F) silicone gel group. Error bars represent SD. A column represents mean ± SD, *n* = 4 **P < 0.01 compared with ART treatment group, ^#^*P* < 0.01 compared with the control group

Figure 8a shows TGF-β1 expression was suppressed in the medium-concentration ART group and silicone gel group when compared to that of the control group. The western blot detection of TGF-β1 protein expression showed a statistical difference between control and treatment groups (**P < 0.01), Fig. 8b. Figure 9a shows SMAD3 expression was suppressed in the medium-concentration ART group and silicone gel group when compared to that of the control group. The Western blot detection of SMAD3 protein expression showed a statistical difference between control and treatment groups (**P < 0.01), Fig. 9b.

## Discussion

Inflammation, granulation, and tissue formation are physiological processes of normal wound healing which occur in sequence. Abnormal reaction in the wound healing process can lead to fibroblast replication with overabundant production of collagen and excessive contraction, resulting in hypertrophic scar (HS) formation [40]. Fibroblasts isolated from HS tissue have been shown to have an increased expression of TGF-β1 and TGF-βR as compared to those from healthy skin and are thought to play a vital role in HS formation [39]. Treatment of HS is typically challenging due to the limited understanding of its pathogenesis [37]. Current therapies for HS include intralesional steroid therapy, pulsed-dye laser ablation, or combining the use of surgery with the above therapies. However, such therapeutic strategies are not satisfactory because they are time consuming or expensive; therefore, the development of novel hypertrophic scar treatments is necessary [38].

Pre-clinical animal models are of great importance for studying the development of hypertrophic scars and evaluating the therapeutic effects of anti-scar treatment. Several models of hypertrophic scarring are reported. The most frequently used hypertrophic scar model is the rabbit ear excisional wound model [4]. In the present study, we successfully established an animal model of hypertrophic scars on rabbit ears by incision wounding, in which each wound ensured removal of full-thickness skin and perichondrium.

Artesunate is a stable derivative of artemisinin which has been declared as antitumor activity in cancer cells and reportedly explains the induction of apoptosis through reactive oxygen species (ROS) generation, angiogenesis inhibitor effect, and inhibition of hypoxia-inducible factor-1α (HIF-1α) by ROS generation [5, 27]. Interestingly, artesunate with a double oxygen bridge structure was discovered as a potential ferroptosis inducer. The study verified that artemisinin derivatives induced iron-dependent cell death (ferroptosis) in tumor cells [22]. Additionally, artesunate targeted the activation of hepatic stellate cell ferroptosis and its effect was associated with the activation of ferritinophagy. At the same time, these findings will provide further mechanisms for understanding the protective effect of artesunate against CCl_4_-induced liver fibrosis [15], the retarded proliferation of lung fibrosis, immunological hepatic fibrosis and induced apoptosis of hepatic stellate cells [3, 37]. Another study displayed that artemisinin derivative dihydroartemisinin specifically caused head and neck cancer cell death through contributing ferroptosis and apoptosis [17]. It reported that ART could decrease the expression of related extracellular matrix proteins and exert an inhibitory effect on cell proliferation [14]. Because of its unique characteristics, ART was recently used to treat proliferative fibrosis diseases, and satisfactory results had been achieved [5]. All these studies prompt ART may play a role in fibrous hyperplastic disease. In the present study, the histopathological assessment showed that ART significantly ameliorated fibroblast/keratinocyte in the dermis. Subsequently, ART decreases Hypertrophic Index, fibroblast number density (NA) and the surface density of collagen fibers in a dose-dependent manner, and thus inhibits fibroblast proliferation, collagen deposition, and scar hyperplasia.

TGF-β1 is a type of cytokine with many biological effects. When tissue is damaged after injury, TGF-β1 is released from inflammatory cells which stimulates fibroblast proliferation, collagen synthesis, deposition and remodeling of ECM [11]. The hypertrophic scar tissue and hypertrophic-derived fibroblast produced more mRNA, protein for TGF-β1 and prolonged expression of the TGF-β receptors than normal skin or fibroblasts derived from normal skin which suggests a possible role for TGF-β1 in hypertrophic scar formation Thus, it can be seen that TGF-β1 has an essential part in serving as a link between injury and fibrosis [7]. It is found that ART could inhibit fibroblast proliferation and reduce epidural fibrosis formation [30]. In the present study, the expression of TGF-β1 was significantly increased in scar tissues, while overexpression of ART could inhibit TGF-β1 expression. Smad signaling transduction proteins act as an intracellular mediator of TGF-β signaling which are the only downstream substrates of TGF-β receptors (TβR) [23]. TGF-β1 may promote the phosphorylation of Smad2 and Smad3 and have a stimulating effect on fibrosis process. Current research suggests that TGF-β/Smad signaling plays a significant role in the pathogenesis of scars. Silencing the Smad2 or Smad3 genes could decrease the collagen synthesis and inhibit fibroblast proliferation [21]. Thus, TGF-/Smad signaling is recognized as a therapeutic target for the treatment of scars. Our study demonstrated that the overexpression of ART significantly inhibited the phosphorylation of Smad2 and Smad3, which is realized by suppressing TGF-β1 expression pathway.

With much research on the mechanisms of scar formation in recent years, many different compounds used TGF-β/SMAD3 as cytokines for the process of hypertrophic scar. TRAP-1-like protein (TLP) is a novel human cytoplasmic protein found associated with the TGF-β/Smad signaling transduction pathway. Besides, it has a significant role such as the ability to regulate Smad2/3 in opposite directions simultaneously. Overexpression of TLP suppresses the expression of the Smad3/4-specific receptor protein (SBE) induced by TGF-β [32]. The study has proved that the mechanism by which pearl powder promotes wound healing is partly due to its ability to stimulate fibroblast mitosis, collagen deposition, and TIMP-1 production [14]. Some preclinical studies have shown that LY2109761 is a TGF-β receptor I (II) kinase inhibitor which is useful as an anticancer compound so medical practitioners have great concern [34]. Subsequently, it reported that LY2109761 attenuated radiation-induced pulmonary murine fibrosis [7]. Our results showed that ART did not cause cell death in HSF cells but moderately decreased the TGF-β1/SMAD3-induced cell proliferation at a medium concentration of ART and silicone gel compared to other concentrations of ART. Further study showed that ART on immunohistochemistry and Western blot analysis significantly inhibits TGF-β1/SMAD3-induced collagen production in HSF cells in a dose-dependent manner and increased expression in control and matrix groups.

In summary, this study provides evidence that overexpression of ART promotes wound healing, alleviates path morphology change in scar tissues, and reduces ECM production by inhibiting the TGF-β/Smad3 signaling pathway in the scaled model of a rabbit. This study also revealed that ART in a dose-dependent manner decreased TGF-β1-/SMAD3-induced proliferation of fibroblasts and collagen production, and attenuated the TGF-β1-/SMAD3-induced contraction of gels containing HSFs. Our results suggest that ART may be a novel therapeutic strategy for hypertrophic scars. Future studies are required to determine other potential mechanisms to develop further and improve the efficacy of ART therapy in the clinical management of hypertrophic scars.

## Abbreviations

ART: Artesunate
AA: Area density of collagen fibers
CASK: Calcium/calmodulin-dependent serine protein kinase
DAB: 3,3-Diaminobenzidine
HI: Hypertrophic Index
H&E: Hematoxylin–eosin staining
ID1: Down-regulation of inhibitors of differentiation 1
NA: Numerical densities of fibroblasts on the area (NA)
OD: Optical density
PBS: Phosphate buffer saline
SDS: Sodium alkyl sulfate
SD: Standard deviation
SMAD3: Small mother against decapentaplegic
TGFβ1: Transforming growth factor
VG: Van Gieson
WB: Western blot
